# Student Carers in Higher Education Institutions: A Scoping Review Protocol

**DOI:** 10.12688/hrbopenres.14047.1

**Published:** 2025-01-23

**Authors:** Maria Pierce, Andrew Darley, Attracta Lafferty

**Affiliations:** 1 Applied Social Studies, Maynooth University, Maynooth, County Kildare, Ireland; 2 University College Dublin School of Nursing Midwifery and Health Systems, Dublin, Leinster, Ireland

**Keywords:** family care, student carers, higher education institutions, policy

## Abstract

Student carers in higher education institutions (HEIs) is an emerging policy issue in many countries. Researchers have sought to synthesise the literature on student carers in HEIs. Runacres *et al.* (2024) conducted a scoping review, which included 14 studies, and Knopf *et al.* (2022) conducted a systematic review of university students who are caring for an older adult, which included six studies. These reviews identified some key themes discussed in the literature. However, there are further areas of discussion that have yet to be explored in the evidence to date. This observation is in the context of a growing body of international literature published on the topic and the greater visibility of family carers post-pandemic. One key area yet to be examined is the policy and practice responses to student carers in HEIs in different countries. The scope of this review will be broader than previous reviews with a specific focus on policy and practice responses regarding this population. This scoping review will: examine definitional issues concerning student carers in HEIs; summarise studies from different countries that provide estimates of the number of student carers in HEIs; identify theoretical perspectives and concepts underpinning research on this topic; and assess the available evidence on the value and outcomes of supports. The 5-stage methodological framework developed by Arksey and O’Malley (2005) will be used to guide this scoping review, as described in detail in this protocol. The findings of the scoping review will be used to inform the enhancement of supports for student carers in HEIs. A range of vehicles will be used to disseminate the findings, including conference presentations, publication in an international peer-reviewed journal, and preparation of a policy brief to disseminate the findings of this scoping review to policymakers and other relevant stakeholders.

## Introduction

### Background and rationale

Student carers in higher education institutes (HEIs) can be defined as any student enrolled in a HEI ‘who cares, unpaid, for a friend or family member who due to illness, disability, a mental health problem or an addiction cannot cope without their support’ ( Carers Trust, 2015: 7). There is a growing body of knowledge and evidence on student carers in HEIs. Researchers have sought to synthesise the literature. Runacres *et al.* (2024) undertook a scoping review to collate, synthesise and map key concepts across the literature. The review, which included 14 studies published up to early 2020, identified the following six themes: impact of caring on student health; impact on university performance, decisions and social life; finance and employment during higher education; impact on study and ability to care; formal supports; and informal supports. Knopf *et al.* (2022) conducted a systematic review of university students caring for an older adult, which included six studies published between 1998 and 2020. The review identified four core themes: student pathways into care; challenges entailed in reconciling the responsibilities of caregiving and studying; impacts of being a student carer and its implications for higher education; and support structures for caregiving students. There are some areas of discussion that have yet to be explored. As student carers in HEIs is an emerging policy issue in Ireland ( Pierce, 2024) and many other counties, the policy and practice responses to student carers in HEIs in different countries is one aspect that needs to be explored and synthesised. Since March 2020, the Covid-19 pandemic has contributed to making carers more visible, including those studying in HEIs. The situation and experience of student carers in HEIs post-pandemic is another aspect that needs to be explored.

The body of international literature published on the topic has grown since May 2020 (e.g. Armstrong-Carter *et al.*, 2022; Chevrier *et al.*, 2023; Day, 2021; Haugland *et al.*, 2020; Munro *et al.*, 2024; Rawlinson, 2024; Taylor *et al.*, 2023; van der Werf *et al.*, 2023). As well as the need to examine policy and practice responses in different countries, there is a need to explore other aspects not covered by existing reviews. In this scoping review, the scope will be extended to examine definitional issues with respect to student carers in HEIs; summarise studies from different countries providing estimates of the prevalence of student carers in HEIs; identify theoretical perspectives and concepts underpinning research on this topic; examine policy responses to student carers in HEIs across different countries; and assess the available evidence on the value and outcomes of supports.

The findings of the proposed scoping review will be used to inform the enhancement of supports for student carers in HEIs. A scoping review has been chosen for this evidence synthesis over other types of literature reviews. The reasons for this are that the review aims to address a broad question on this topic and examine the extent, range and nature of research studies on this topic. It seeks to include studies conducted on this topic using many different study designs, but it is not intended to assess the quality of included studies.

### Research aims and objectives

The overall aim of the scoping review is to collate and synthesise what is known from the existing literature about student carers in HEIs. A key focus will be on the policy and practice responses to student carers in HEIs in different countries. The scoping review has several objectives, which are to identify: how student carers in HEIs are typically defined in policy, research and practice; what methodologies are used in research on student carers in HEIs; which theoretical perspectives and concepts underpin research students on student carers in HEIs; estimates of the prevalence of student carers in HEIs and methods used in producing these estimates; the main characteristics of student carers in HEIs; the main impacts of student caring in HEIs for students and staff; and the evidence that exists on approaches developed to identify and support student carers in HEIs.

Larkin and Kubiak (2021: 142) propose adopting the term ‘caring-experienced students’ (not to be confused with care-experienced students) as it is inclusive of ‘students who are, become or have been a carer’ and recognises the several vulnerabilities that all these students are likely to experience. This scoping review adopts the term ‘caring-experienced students’ to mean students in HEIs who are, become or have been caring, unpaid, for a friend or family member who due to illness, disability, a mental health problem or an addiction cannot cope without support. In our use of the term, we include students who are caring for a sibling where a family member is experiencing a physical or mental health problem and students who are parents caring for a child with a chronic illness or mental health problem, but not caregiving for children more broadly.

## Methods

The scoping review is informed by the methodological framework developed by Arksey and O’Malley (2005), which proposes a five stage framework. Hence, the review process follows the following five stages:

- Stage 1: Identifying the research question
- Stage 2: Identifying relevant studies
- Stage 3: Study selection and screening
- Stage 4: Charting the data and data extraction
- Stage 5: Collating, summarising and reporting the results

The approach that will be taken to each of these stages is outlined in greater detail below. A consultation exercise will not be conducted as part of the review, although consultation with a group of student carers or with access/support staff in HEIs will be considered for any future research projects on this topic.

The authors used the guidance and template developed by Lely *et al.* (2023) and the Preferred Reporting Items for Systematic reviews and Meta-Analyses extension for Scoping Reviews (PRISMA-ScR) ( Tricco *et al.*, 2018) for writing this scoping review protocol. The PRISMA ScR ( Tricco *et al.*, 2018) is the reporting guideline that will be used for writing the final scoping review manuscript.

### Stage 1: identifying the research question

The first methodological stage when undertaking a scoping review is to determine the focus of the research questions ( Arksey & O’Malley, 2005). Following a preliminary review of the literature, the research team has identified an overall research question to guide the scoping review, as follows: What is known from the existing literature about student carers in higher education institutions, with a key focus on the policy and practice responses to student carers in different countries? The following research questions will also guide the scoping review:

- What definitions of student carers in HEIs are typically adopted?
- What estimates of the number/proportion of student carers in HEIs have been produced, and what methods are used for producing estimates?
- What are the main socio-demographic characteristics of student carers in HEIs?
- What theoretical perspectives and concepts underpin research studies on student carers in HEIs?
- What type of studies have been conducted and what methods have been used?
- What are the experiences and impacts of student caring in HEIs for students and staff?
- What evidence exists on approaches developed to identify and support student carers in HEIs?

### Stage 2: identifying relevant studies

For the second stage of the scoping review, the authors will develop a search strategy for the purposes of identifying all relevant literature related to student carers in higher education. Relevant literature includes publications that have a principal focus on evidence regarding student carers attending higher level education. Sources of evidence will be peer-reviewed empirical studies (qualitative, quantitative or mixed methods), reviews, conference abstracts, book chapters as well as grey literature. The author team will use a three-step search strategy in this scoping review. This follows the recommendations by the Joanna Briggs Institute (JBI) Manual for Evidence Synthesis ( Peters *et al.*, 2020). First, the author team will conduct an initial search of two databases, i.e., Applied Social Sciences Index & Abstracts (ASSIA) and Education Resources Information Centre (ERIC). Second, they will conduct an analysis of the text words contained in the title and abstract of retrieved papers, and of the index terms used to describe the articles. The author team will use the ‘PCC’ mnemonic (i.e., population, concept and context) to frame the research question and the subsequent search. In the current review, this refers to:

Population: Students who are attending higher level education while also providing unpaid care
Concept: Caring for a family member while studying within higher level education
Context: Higher Education Institutions

The second step entailing a second search using all identified keywords and index terms will then be undertaken across all included databases. Search terms will focus on two core concepts: "students caring for a family member" and "higher education". Databases to be searched include MEDLINE, ERIC, PsycINFO, Cumulated Index in Nursing and Allied Health Literature. (CINAHL), ASSIA and the British Education Index. The search will also include synonyms and related terms to ensure comprehensive coverage. Boolean operators (AND, OR) and truncation (*) will be applied to refine the search. Based on this exploratory scoping phase, the search strings for each database will be finalised. Database searches are intended to commence in January 2025. The proposed search terms which will be used to develop the search strategy are listed in Table 1.

**Table 1.** Search terms.

PCC	Concept	Search Terms
Population	Family	“Famil*” OR "Family"[Mesh] OR “sibling*” OR "Siblings"[Mesh] OR “spous*” OR "Spouses"[Mesh] OR “relative*” OR “wife” OR “wives” OR “husband*” OR “partner*” OR “significant other” OR “next of kin” OR “kinship” OR “father” OR “mother” OR “daughter*” OR “son*” OR “child*” OR “brother*” OR “sister*” OR “informal” OR “unpaid” OR “non-professional*” OR “parent*” OR "Parents"[Mesh]
	**AND**	
	Carer	“Care*” OR “caregiver*” OR “care-giver*” OR “caregiving” OR “care-giving” OR “caring” OR “unpaid care*” OR “Family care” OR "Caregivers"[Mesh]) OR “eldercare” OR “elder-care” OR “elder care”
Concept	Student	"student*" OR "Students"[Mesh] OR “young adult*” OR “Young Adult"[Mesh] OR “undergrad*” OR “postgrad*” OR “‘graduate” OR “graduand*” OR freshman* OR “sophomore*” OR “scholar*” OR “registrant*” OR “doctora*” OR “phd*” OR “master*” OR “College Senior*” OR “Two Year College Student*”
Context	Higher Education	"higher education" OR "HEI" OR "university" OR "college" OR "tertiary education" OR "postsecondary education" OR “institut*” OR “education” OR “post-18 education” OR “third level” OR “higher level education” OR “academic institution*” OR "Education, Graduate"[Mesh] OR "Universities"[Mesh]

The third step will involve hand searching of the reference lists of identified reports and articles to identify any additional sources. Where it is deemed necessary, the reviewers will contact authors of primary articles or reviews for further information. The reviewers will also undertake a search for grey material, such as unpublished work, conferences, reports, and website information. Each database search will combine terms related to both the student carer role and higher education institutions. Grey literature search will be also performed using the term ‘student carer’ in a range of education-related evidence sources and data-hubs linked to relevant family carer organisations such as The Carer’s Trust and Eurocarers. Additionally, the following key journals will be hand-searched to identify articles that have been missed through the electronic database search: *International Journal of Inclusive Education; Journal of Further and Higher Education; Higher Education, Research & Development; International Journal of Care and Caring.*

The full search strategy for at least one of the major databases used will be included as an appendix to the scoping review manuscript ( Tricco *et al.*, 2018).

### Stage 3: study selection and screening

Before commencing screening of the evidence found from the search strategy, the author team will discuss and decide on the inclusion criteria to ensure that all team members have a shared interpretation of the criteria. In line with the methodological guidance from Arksey and O’Malley (2005), the final inclusion and exclusion criteria will be refined based on increasing familiarity with the body of literature and type of data available. The authors seek to restrict the synthesise to the last 25 years of evidence on this topic, because of the dearth of evidence prior to the year 2000, as highlighted by Runacres *et al.* (2024). Table 2 presents the initial inclusion criteria for the scoping review. The full list of eligibility criteria (e.g., the search period, language, and publication status) and rationale will be provided in the scoping review manuscript ( Tricco *et al.*, 2018).

**Table 2.** Inclusion criteria.

Inclusion Criteria	Exclusion Criteria
1. Studies focusing on students in higher education with caregiving responsibilities. 2. Research on the impact of caregiving on students’ academic performance, well-being, institutional experiences or policy/practices. 3. Studies addressing interventions, support mechanisms, or policies aimed at student carers. 4. Research articles, reviews, conference abstracts, working papers, reports, books and book chapters. 5. Written in English (the language understood by the authors). 6. Published between 2000 and 2024.	1. Studies focusing solely on carers outside higher education (e.g., adult carers not in higher educational settings). 2. Research focusing on student carers in secondary or primary school. 3. Studies that do not include a clear focus on student caregiving or higher education institutions. 4. Not written in English.

Once the searches have been completed within all relevant databases, retrieved articles will be imported into the reference management software, Endnote 21. To ensure that each study retrieved is not repeated, duplicate records will be identified and removed within Endnote. This will provide a more reliable final set of studies. For the purposes of screening of the retrieved literature, this set of articles will be imported into the systematic review software tool, Covidence ( www.covidence.org). Using this software, the reviewers will conduct a final check to ensure that remaining duplicate studies have been identified and removed. Pilot-testing of the screening process will then be conducted using Covidence. This will involve selecting a random sample (e.g., n=25) of the articles retrieved. This will be followed by screening of each article, based on title and abstract, which will be conducted independently by two team members. The independent review is aimed at reducing screening bias. The purpose of the screening is to determine if an article is to be included or excluded and will use the pre-determined inclusion and exclusion criteria. Following the title and abstract screening, the included articles will be subjected to full-text screening, conducted independently by two reviewers. If a consensus regarding inclusion and exclusion is not reached between the two reviewers, this will be resolved by the decision of a third team member. The process of study selection will be reported using the PRISMA-ScR guidelines. Once the review is completed, the reporting of this process will be updated and provided in the scoping review manuscript ( Tricco *et al.*, 2018).

### Stage 4: data charting/collection/extraction

The author team will develop and agree on an Excel data extraction template and the extraction variations, and the studies retrieved through the search and screening process that meet the inclusion criteria will be inputted in the spreadsheet. The type of data that will be extracted will help to answer the scoping review’s overall research question and objectives. These include author(s), year of publication, country of publication, disciplinary background, definition of ‘student carer’, theoretical frameworks, study aims, study design, methods adopted, type of data collected, study population, and main findings. The rules for data extraction will be agreed by the author team and stored in a shared folder. The methods of charting, collecting and extracting data will be fully described in the scoping review manuscript ( Tricco *et al.*, 2018). 

### Stage 5: collating, summarising and reporting the results

The data extracted will be collated, synthesised and presented numerically and thematically in a meaningful way (e.g., using tables, figures, diagrams). A PRISMA flow diagram will be used to provide a visual overview, depicting the screening process and showing the number of sources of evidence reviewed, assessed for eligibility and included in the review, and the number excluded at each stage. The characteristics regarding the sources of evidence and results of individual sources of data will be presented ( Tricco *et al.*, 2018). Using reflexive thematic analysis, the paper will be summarised in a way that answers the scoping review’s research questions ( Braun & Clarke, 2019). For the purposes of validation, a second author team member will review and check the results. The limitations of the scoping review process will be reported ( Tricco *et al.*, 2018).

## Dissemination

By summarising in detail the findings and range of research on the topic of student carers in HEIs, this scoping review will provide a mechanism for disseminating key research findings to a range of relevant stakeholders including policy makers, HEIs, student unions, community and voluntary sector organisations with an interest in family carers, and student carers, who might otherwise lack time or resources to undertake such work themselves.

It is intended to present the findings at relevant conferences and to publish the findings of this scoping review in an international peer-reviewed journal, e.g. International Journal of Inclusive Education or Journal of Further and Higher Education. It is intended to prepare a policy brief to disseminate the findings of this scoping review to policymakers and other stakeholders. 

## Conclusion

The primary objective of this scoping review is to build on existing reviews of student carers in HEIs and address areas of discussion not yet covered. It will collate, summarise and map what is known from the existing literature about student carers in higher education institutions, with a particular focus on the policy and practice responses. Other areas to be addressed include: examining definitional issues with respect to student carers in HEIs; summarising studies from different countries providing available estimates of the number of students carers in HEIs; identifying theoretical perspectives and concepts underpinning research on this topic; and providing evidence that exists on approaches developed to identify and support student carers in HEIs. This scoping review will thus provide updated and valuable information for dissemination to a wide range of relevant stakeholders including policy makers and HEIs.

## Ethics

Ethical approval is not required for this evidence synthesis study.

## Data Availability

No data are associated with this study.

## Extended data

No extended data are associated with this study.

## References

[ref-1] Arksey H O'Malley L : Scoping studies: towards a methodological framework. *Int J Soc Res Methodol.* 2005;8(1):19–32. 10.1080/1364557032000119616

[ref-2] Armstrong-Carter E Panter AT Hutson B : A university-wide survey of caregiving students in the US: individual differences and associations with emotional and academic adjustment. *Humanit Soc Sci Commun.* 2022;9(1): 300. 10.1057/s41599-022-01288-0

[ref-3] Braun V Clarke V : Reflecting on reflexive thematic analysis. *Qual Res Sport Exercise Health.* 2019;11(4):589–597. 10.1080/2159676X.2019.1628806

[ref-4] Carers Trust: Supporting students with caring responsibilities: ideas and practice for universities to help student carers access and succeed in Higher Education.London: Carers Trust,2015. Reference Source

[ref-5] Chevrier B Untas A Dorard G : Young adult caregivers in Higher Education: a study of prevalence in France. *J Further High Educ.* 2023;47(5):699–710. 10.1080/0309877X.2023.2190878

[ref-6] Day C : An empirical case study of young adult carers' engagement and success in Higher Education. *Int J Inclus Educ.* 2021;25(14):1597–1615. 10.1080/13603116.2019.1624843

[ref-7] Haugland BSM Hysing M Sivertsen B : The burden of care: a national survey on the prevalence, demographic characteristics and health problems among Young Adult Carers attending Higher Education in Norway. *Front Psychol.* 2020;10:2859. 10.3389/fpsyg.2019.02859 32038347 PMC6989434

[ref-8] Knopf L Wazinski K Wanka A : Caregiving students: a systematic literature review of an under-researched group. *J Further High Educ.* 2022;46(6):822–835. 10.1080/0309877X.2021.2008332

[ref-9] Larkin M Kubiak C : Carers and Higher Education: where next? *Widen Participat Lifelong Learn.* 2021;23(2):130–151. 10.5456/WPLL.23.2.130

[ref-10] Lely J Morris HC Sasson N : How to write a scoping review protocol: guidance and template. *OSF*,2023. 10.17605/OSF.IO/YM65X

[ref-11] Munro D Willis J Gibson A : From inside the head to putting it on the table: supporting reflexive decision-making for unpaid female carers considering Higher Education. *Reflect Pract.* 2024;25(3):426–440. 10.1080/14623943.2024.2321501

[ref-12] Peters MDJ Marnie C Tricco AC : Updated methodological guidance for the conduct of scoping reviews. *JBI Evid Synth.* 2020;18(10):2119–2126. 10.11124/JBIES-20-00167 33038124

[ref-13] Pierce M : Student carers in Higher Education institutions in Ireland: an emerging policy issue.Dublin: UCD Geary Institute for Public Policy,2024. Reference Source

[ref-14] Rawlinson S : 'Little Islands': challenges and opportunities for student carers in Higher Education. *Int J Inclus Educ.* 2024;1–16. 10.1080/13603116.2024.2430543

[ref-15] Runacres J Herron D Buckless K : Student carer experiences of Higher Education and support: a scoping review. *Int J Inclus Educ.* 2024;28(7):1275–1292. 10.1080/13603116.2021.1983880

[ref-16] Taylor J Gleeson P Teague T : Practices of inclusion for carers who are Higher Education students. *Int J Inclus Educ.* 2023;27(13):1469–1486. 10.1080/13603116.2021.1900424

[ref-17] Tricco AC Lillie E Zarin W : PRISMA extension for scoping reviews (PRISMA-ScR): checklist and explanation. *Ann Intern Med.* 2018;169(7):467–473. 10.7326/M18-0850 30178033

[ref-18] van der Werf HM Paans W Francke AL : Identifying and supporting students with a chronically Ill family member: a mixed-methods study on the perceived competences and role views of lecturers. *Int J Environ Res Public Health.* 2023;20(6):4978. 10.3390/ijerph20064978 36981887 PMC10049440

