# Supplementary material for: Student Carers in Higher Education Institutions: A Scoping Review Protocol
Source: HRB Open Res. 2025 Jan 23;8:9. [Version 1] doi: 10.12688/hrbopenres.14047.1 (PMC11880753; doi:10.12688/hrbopenres.14047.1)
Supplement: Peer review history

# Reviewer report: Reviewer response for version 1

**DOI:** 10.21956/hrbopenres.15424.r45954

**Published:** 2025-03-04

**Authors:** Peter Unwin

**Affiliations:** 1 University of Worcester, Worcester, UK

This is a well-written proposal on an important issue that has not been fully addressed by previous research.

 The structure flows well and it would appear that key extant literature is already known to the authors, who set out an ambitious project.

 It will be challenging to compare different countries' experiences, and to make reliable comparators. Contextual  factors such as levels of fees / complexities in any 'carers' allowances' and percentages of 'study at home' / flexi studies in different disciplines will be very different across countries. Depending on how manageable the logistics appear, it might be worth narrowing the scope to specific disciplines which might allow for more validity comparisons to be drawn. For example, some disciplines ( history/ languages, literature) lend themselves more to flexi - hours whereas certain science subjects demand on-campus lab time and vocational courses such as nursing and social work have considerable placement demands, with very little in-built flexibility. It may , of course, be that the team find that the study foci of caring-experienced students reflect a tendency toward certain subjects.

 Another factor of interest, which might be a challenge too far, is to consider cohorts which became carers after their studies had commenced as compared to those who applied to HEIs when they were already carers.

 On a point of detail, I note the team's preference for the term 'caring-experienced students' but that the proposed research questions use the term 'student carers'.

Is the study design appropriate for the research question?

Yes

Is the rationale for, and objectives of, the study clearly described?

Yes

Are sufficient details of the methods provided to allow replication by others?

Yes

Are the datasets clearly presented in a useable and accessible format?

Not applicable

Reviewer Expertise:

Voices of service users and carers in education -Marginalised groups - Social Inclusion- Disability

I confirm that I have read this submission and believe that I have an appropriate level of expertise to confirm that it is of an acceptable scientific standard.

# Reviewer report: Reviewer response for version 1

**DOI:** 10.21956/hrbopenres.15424.r45189

**Published:** 2025-03-03

**Authors:** Mary Larkin

**Affiliations:** 1 The Open University, Milton Keynes, UK

The authors are to be congratulated on their use of such an Inclusive definition of Student Carers in Higher Education Institutions, Rigorous use of Arksey and O’Malley’s five stage framework. They also present some excellent ideas about ways of accessing and including grey literature.

 If the aims of the scoping review can achieved it will certainly address key outstanding issues which underpin future student carer research and policy making

 There are a couple of weaknesses::

 - It is not clear how they will be using their inclusive definition of a student carer throughout the scoping - review

 It will be worth working with carers organisations on the dissemination and eliciting their support in working with policy makers

Is the study design appropriate for the research question?

Yes

Is the rationale for, and objectives of, the study clearly described?

Yes

Are sufficient details of the methods provided to allow replication by others?

Yes

Are the datasets clearly presented in a useable and accessible format?

Yes

Reviewer Expertise:

care, carers and caring

I confirm that I have read this submission and believe that I have an appropriate level of expertise to confirm that it is of an acceptable scientific standard.

# Reviewer report: Reviewer response for version 1

**DOI:** 10.21956/hrbopenres.15424.r45182

**Published:** 2025-02-26

**Authors:** Jessica Runacres

**Affiliations:** 1 University of Staffordshire, Stoke-on-Trent, UK

This is a protocol for a scoping review focusing on student carers in higher education which is an emerging and interesting area of research. The researchers present a detailed outline of the methods to be undertaken and have generated broad research questions which address a noted gap in the current literature. It is clear that a significant amount of thought has been put into the proposed methods which are well articulated and suitably referenced. Some points to consider below:

 The work of Runacres et al. (2024) and Knopf et al. (2022) is mentioned in the introduction, including the findings, and it is stated that there are some areas of discussion that have yet to be explored, however specifically how this scoping review will build on this existing work is unclear. Some further critical engagement with the existing literature reviews on this topic would be beneficial here. Furthermore, the introduction argues that “there is a need to explore other aspects not covered by existing reviews” but why could be further justified.

 There is a brief argument for the use of a scoping review at the end of the introduction, this argues that this scoping review intends to address a broad question and incorporate studies using a variety of different study designs, however, this is possible within a systematic review. This section also states that the reviewers are using a scoping review as they do not intend to assess quality, and, whilst this is the case within a scoping review, it should not be used as an argument for the use of this. Instead, it would be interesting to hear why it is important that quality is not assessed for this review. Also, it is stated in this protocol that policy responses to student carers in HEIs will be examined. I would assume that this information is within the grey literature, which therefore means that a scoping review would be suitable, and quality appraisal would be challenging. This justification for the chosen review approach could be mentioned here.

 It is mentioned at the end of the introduction that the term “caring-experienced students” will be adopted by this review, however this is not the case, instead “student carer” is used throughout. This needs to be clarified.

 The aim and objectives are clear, although perhaps very broad for a single review. I am unsure how you will be able to recognise these broad objectives within a single analysis.

 There is overlap in your search terms. For example, al the subsequent terms would be captured by the first ““Care*” OR “caregiver*” OR “care-giver*” OR “caregiving” OR “care-giving” OR “caring”. This is the case within other levels of your search strategy.

 Your definition for this review of student carers is those who provide care for a friend or family member, yet “friend” is not encompassed within your search terms.

Is the study design appropriate for the research question?

Yes

Is the rationale for, and objectives of, the study clearly described?

Partly

Are sufficient details of the methods provided to allow replication by others?

Yes

Are the datasets clearly presented in a useable and accessible format?

Not applicable

Reviewer Expertise:

Student carers, informal carers, literature reviews

I confirm that I have read this submission and believe that I have an appropriate level of expertise to confirm that it is of an acceptable scientific standard, however I have significant reservations, as outlined above.
